# Comparison of levocetirizine pharmacokinetics after single doses of levocetirizine oral solution and cetirizine dry syrup in healthy Japanese male subjects

**DOI:** 10.3109/21556660.2014.928302

**Published:** 2014-06-03

**Authors:** Hiroko Ino, Katsutoshi Hara, Gosuke Honma, Yohei Doi, Hiroyuki Fukase

**Affiliations:** 1 Medicines Development (Clinical Pharmacology), Development & Medical Affairs Division, GlaxoSmithKline K.K., TokyoJapan; 2 Biomedical Data Sciences Department, Development & Medical Affairs Division, GlaxoSmithKline K.K., TokyoJapan; 3 Medicines Development (Clinical Pharmacology), Development & Medical Affairs Division, GlaxoSmithKline K.K., TokyoJapan; 4 CPC Clinical Trial Hospital, Medipolis Medical Research Institute, KagoshimaJapan

**Keywords:** Levocetirizine, Cetirizine, Pharmacokinetics, Japanese

## Abstract

**Objective:**

Levocetirizine, the R-enantiomer of cetirizine, is classified as a second generation antihistamine used for the treatment of allergic disorders. This study aimed to compare exposure to levocetirizine when given as levocetirizine oral solution (OS) 5 mg to that when given as cetirizine dry syrup (DS) 10 mg, which contains equal proportions of levocetirizine and dextrocetirizine, in healthy Japanese male subjects.

**Methods:**

The study was conducted in an open-label, single dose, randomized and two-way cross-over design. Eligible subjects were allocated to one of two groups and received either levocetirizine OS 5 mg or cetirizine DS 10 mg under fasting conditions, and the alternate treatment after a 7-days washout period. Serial blood samples were taken after each administration, and plasma levocetirizine concentrations were determined using a validated LC-MS/MS method. Pharmacokinetic parameters were calculated by using non-compartmental analysis. Comparisons of levocetirizine pharmacokinetics were conducted with maximum concentration (*C*_max_) and the area under the plasma concentration-time curve from dosing until 48 h post-dose (AUC_0–48_) after each treatment.

**Clinical Trial registration number:**

ClinicalTrials.gov identifier is NCT01622283

**Results:**

The mean *C*_max_ and AUC_0–48_ of levocetirizine after a single dose of levocetirizine OS 5 mg and cetirizine DS 10 mg were 203.3 ± 42.49 ng/mL and 1814.9 ± 304.22 ng.hr/mL, and 196.5 ± 31.31 ng/mL and 1710.5 ± 263.31 ng hr/mL, respectively. The ratios and the 90% CIs of the geometric least squares means ratios of *C*_max_ and AUC_0–48_ were 1.027 (0.968–1.091) and 1.059 (1.024–1.094), respectively.

**Limitation:**

The small sample size and single dose design of this study prevent definitive conclusions regarding the pharmacokinetics and safety of levocetirizine OS in a Japanese patient population being made. Study limitations include conducting the study in adult males, not in children.

**Conclusions:**

Levocetirizine exposure in plasma was equivalent when given as levocetirizine OS 5 mg and as cetirizine DS 10 mg. Both preparations were safe and well-tolerated in healthy Japanese male subjects.

## Introduction

Levocetirizine hydrochloride (hereafter, levocetirizine) is one of the two enantiomers (R-enantiomer: levocetirizine, S-enantiomer: dextrocetirizine) of cetirizine hydrochloride (hereinafter, cetirizine). Levocetirizine is classified as a second generation antihistamine and is available for the treatment of allergic disorders, such as allergic rhinitis and chronic idiopathic urticaria^1^. The antihistaminic activity of cetirizine is primarily due to levocetirizine^2^, which has high affinity and selective antagonistic activity against histamine (H_1_) receptors and inhibits eosinophil chemotaxis^3,4^. A large number of clinical studies have demonstrated the efficacy, tolerability, long-term safety, and patient satisfaction of levocetirizine^5–7^. Levocetirizine has been reported to be rapidly and extensively absorbed following oral administration of levocetirizine 5 mg and 10 mg as a tablet formulation in healthy Japanese male subjects, where time to reach the maximum concentration (*t*_max_) was achieved between 0.8–1 h after administration and declined with the terminal half-life (*t*_1/2_) of 7.3–7.6 h^8^. Levocetirizine is eliminated predominantly by renal excretion, with limited metabolism^9^.

Levocetirizine is available only as a 5 mg oral tablet and is used in adults and children aged 7–14 years in Japan while cetirizine dry syrup (DS) has been marketed since 2009 and has proven a preferred dosage form for children below the age of 6 years^10^. When used in pediatric patients under the age of 15 years with allergic disorders, a levocetirizine 5 mg oral tablet must be divided in half. Due to the prevalence of allergic disorders in pediatric patients, a new formulation of second generation antihistamines that can be easily taken by pediatric patients is highly desirable, such as oral solution (OS) and DS^11^.

The objective of this study was to compare exposure to levocetirizine when given as levocetirizine OS 5 mg and as cetirizine DS 10 mg, the latter of which contains equal proportions of levocetirizine and dextrocetirizine, in healthy Japanese male subjects.

## Subjects and methods

### Subjects

The key inclusion criteria were as follows: age 20–55 years with body mass index (BMI) in the range 18.5–24.9 kg/m^2^ and good general health as determined by medical history, clinical examination, 12-lead electrocardiogram (12-lead ECG) and clinical laboratory tests. The screening procedure was conducted within 30 days prior to the first dosing.

### Study design

The study was conducted in an open-label, single dose, randomized and 2-way cross-over design. Eligible subjects were allocated to one of two groups. Each participant received one treatment of either levocetirizine OS 5 mg or cetirizine DS 10 mg under fasting conditions and received the alternate treatment after a 7-days washout period. Ten milliliters of levocetirizine 0.5 mg/mL oral solution (lot no. 623C22, GlaxoSmithKline K.K., Japan) containing 5 mg of levocetirizine was administered with 140 mL of water. 0.8 g of cetirizine dry syrup 1.25% (lot no. 623C21, GlaxoSmithKline K.K., Japan) containing 10 mg of cetirizine was dissolved in 100 mL of water, and was administered with 50 mL of water. All participants stayed at the unit until the completion of pharmacokinetic and safety assessments 48 h post-dose, and returned to the unit 7 days after the second dose for follow-up assessment.

The study was undertaken at CPC Clinical Trial Hospital, Medipolis Medical Research Institute (Kagoshima, Japan) in compliance with the Good Clinical Practice Guidelines after the protocol was approved by CPC Clinical Trial Hospital Institutional Review Board. Written informed consent was obtained from each subject in accordance with the Declaration of Helsinki.

### Assessment of plasma levocetirizine concentrations

Blood samples were collected into EDTA tubes pre-dose and at 0.25, 0.5, 1, 1.5, 2, 3, 4, 6, 9, 12, 16, 24, 36, and 48 h post-dose. Plasma samples were prepared within 60 min of blood collection and kept frozen below −20°C until analysis. Levocetirizine concentrations in plasma were determined by using an LC-MS/MS method validated at Shin Nippon Biomedical Laboratories, Ltd (Japan) based on a previously reported method^12^ with slight modification: less plasma volume and different calibration range. Levocetirizine was extracted by liquid–liquid extraction after protein precipitation of 100 μL of human plasma containing an isotopically labelled internal standard (Cetirizine-d8 dihydrochloride). Extracts were analyzed by an LC-MS/MS system (API3000, AB Sciex, Japan) using an APCI interface and multiple reaction monitoring, and transition mass for levocetirizine and internal standard were 389.1–201.1 and 397.2–201.1, respectively. The calibration range was 2–1000 ng/mL with 1/x weighted linear regression, and the correlation of coefficient of the curves were greater than 0.9900. The precision (CV [%]) and accuracy (bias [%]) obtained in the method were both less than 15%.

### Pharmacokinetic and statistical analysis

Pharmacokinetic parameters were calculated from the plasma levocetirizine concentrations and actual sampling times with standard non-compartmental methods using Phoenix® WinNonlin® version 6.3 (Certara USA, Inc., St Louis, MO). *C*_max_ and *t*_max_ were obtained directly from the data. The terminal rate constant (*k*_el_) was obtained by regression analysis of the terminal log-linear part of the concentration-time curve for each subject. *t*_1/2_ was calculated as 0.693/*k*_el_. The area under the plasma concentration-time curve from dosing until 48 h post-dose (AUC_0–48_) was calculated with the linear trapezoidal rule. AUC from dosing to infinity (AUC_0–∞_) was calculated as AUC_0–_*_t_* + *C_t_*/*k*_el_, where AUC_0–_*_t_* and *C_t_* are AUC from dosing to time of last quantifiable plasma concentration and the last observed quantifiable concentration, respectively. The percentage of the extrapolation from AUC_0–_*_t_* to AUC_0–∞_ (%AUCex) was calculated as [AUC_0–∞_ − AUC_0–_*_t_*]/AUC_0–∞_ × 100.

For each of the derived levocetirizine pharmacokinetic parameters, summary statistics were calculated by treatment. The primary endpoints used to compare exposure to levocetirizine when given as levocetirizine OS 5 mg and as cetirizine DS 10 mg were AUC_0–48_ and *C*_max_. After log*_e_*-transformation, AUC_0–48_ and *C*_max_ given as levocetirizine OS 5 mg or cetirizine DS 10 mg were analyzed separately by analysis of variance (ANOVA) with parameters for treatment and group as fixed effects, and subjects within group as random effect. Point estimates and associated 90% confidence intervals (CIs) for the difference of two regimens [logμ_test_ − logμ_ref_] were constructed using the residual variance where μ_test_ and μ_ref_ are the geometric mean AUC_0–48_ or *C*_max_ of levocetirizine oral solution 5 mg and cetirizine DS 10 mg, respectively. These estimate values were then exponentially back-transformed to provide point estimates and associated 90% CIs for the geometric mean ratio [μ_test_/μ_ref_]. Levocetirizine exposures in plasma were considered to be equivalent between two preparations when the 90% CIs of the geometric mean ratio of the plasma levocetirizine AUC_0–48_ and *C*_max_ were within the range ‘0.80–1.25’, referring to the Japanese Guideline for Bioequivalence Studies of Generic Products^13^.

Sample size calculation was based on the within-subject estimates of variability (CVw%). Assuming CVw% of 20% conservatively, it was estimated that sample size of 19 subjects were to provide at least 90% power to demonstrate bioequivalence. This calculation was based on a two-side *t*-test (TOST) procedure with each type 1 error rate of 5% and assumed a true ration geometric mean ratio of 1.00. This procedure was corresponding to acceptance criteria for 90% confidence interval.

### Safety and tolerability assessment

Adverse events were recorded throughout the study. Other safety assessments included clinical laboratory tests, vital signs and 12-lead ECG. AEs were planned to be coded with the Medical Dictionary for Regulatory Activities (MedDRA) version 15.0. SAS version 9.2.2 (SAS® Institute Inc., Cary, NC) was used to create datasets and perform statistical analyses.

## Results

### Subjects

Twenty eligible subjects were enrolled in the study and all subjects completed the study. All participants were healthy Japanese adult males, aged 20–48 years (mean ± SD, 28.7 ± 8.1 years). Body weight and BMI were 51.3–79.4 kg (mean ± SD, 63.9 ± 7.1 kg) and 18.5–24.1 kg/m^2^ (mean ± SD, 21.6 ± 1.9 kg/m^2^), respectively.

### Pharmacokinetics

**Figure 1.**
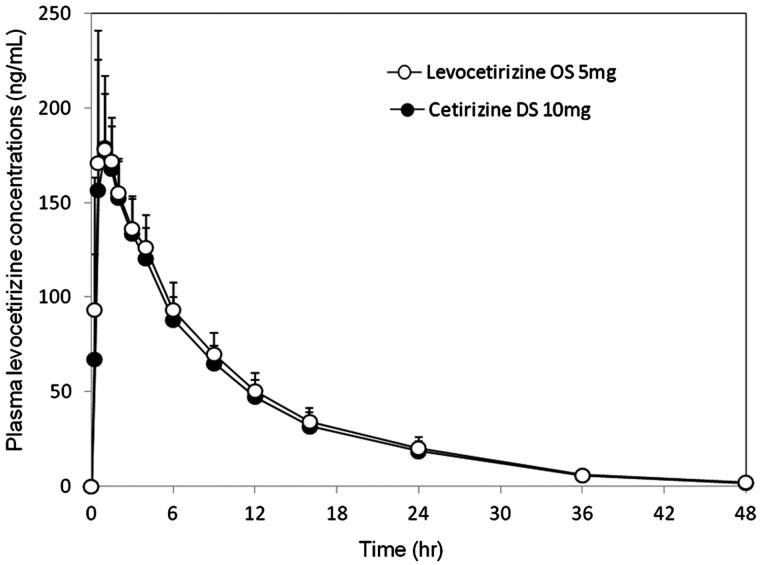
Mean (+SD) plasma concentration-time plots of levocetirizine after single dose of levocetirizine OS 5 mg (○) and cetirizine DS 10 mg (•) under the fasting condition in 20 healthy Japanese male subjects. OS, oral solution; DS, dry syrup; hr, hour.

Mean plasma levocetirizine concentration-time profiles and levocetirizine pharmacokinetic parameters after single oral dosing of levocetirizine OS 5 mg and cetirizine DS 10 mg are shown in Figure 1 and Table 1, respectively. Levocetirizine was absorbed rapidly following both treatments, with *t*_max_ ranging from 0.50–1.50 h post-dose in the fasted state. These concentrations then declined with a mean *t*_1/2_ of approximately 7.9 h.

**Table 1.** Plasma levocetirizine pharmacokinetic parameters following a single oral administration of levocetirizine OS 5 mg and cetirizine DS 10 mg in 20 healthy Japanese male subjects.

Treatment	*C*_max_ (ng/mL)	*t*_max_ (h)	AUC_0–48_ (ng.h/mL)	AUC_0–∞_ (ng.h/mL)	%AUCex (%)	*t*_1/2_ (h)
Levocetirizine OS 5 mg	203.3 (42.49)	0.75 (0.50–1.50)	1814.9 (304.22)	1844.7 (317.56)	2.19 (0.77)	7.91 (1.00)
Cetirizine DS 10 mg	196.5 (31.31)	1.00 (0.50–1.50)	1710.5 (263.31)	1737.1 (278.99)	2.35 (0.71)	7.85 (1.00)

All parameters are reported as arithmetic mean (SD), except *t*_max_ as median (range). OS, oral solution; DS, dry syrup; *C*_max_, maximum concentration; *t*_max_, time to reach the maximum concentration; AUC_0–48_, plasma concentration-time curve from dosing until 48 h post-dose; AUC_0–∞_, AUC from dosing to infinity; %AUCex, percentage of the extrapolation from AUC_0–_*_t_* to AUC_0–∞_; *t*_1/2_, terminal half-life; h, hour.

The ratios and 90% CIs of the geometric least squares mean for levocetirizine *C*_max_ and AUC_0–48_ values following administration of levocetirizine OS 5 mg or cetirizine DS 10 mg are shown in Table 2. The ratios and the 90% CIs of the geometric mean ratio of *C*_max_ and AUC_0–48_ were 1.027 (0.968–1.091) and 1.059 (1.024–1.094), respectively.

**Table 2.** Comparison of the geometric least squares mean ratio and 90% confidence interval of AUC_0-48_ and C_max_ for levocetirizine OS 5 mg and cetirizine DS 10 mg in twenty healthy Japanese male subjects.

Pharmacokinetic parameter	Geometric LS mean		Ratio* of geometric LS mean	90% CI of ratio*
	Levocetirizine OS 5 mg	Cetirizine DS 10 mg		
C_max_ (ng/mL)	199.57	194.27	1.027	0.968–1.091
AUC_0–48_ (ng h/mL)	1791.58	1692.55	1.059	1.024–1.094

*Ratio = Geometric LS mean of levocetirizine oral solution 5 mg/Geometric LS mean of cetirizine dry syrup 10 mg. OS, oral solution; DS, dry syrup; LS mean, Least Square mean; *C*_max_, maximum concentration; AUC_0–48_, plasma concentration-time curve from dosing until 48 h post-dose; CI, confidence interval; h, hour.

### Safety and tolerability

No participants experienced AEs or clinically significant findings in clinical laboratory tests, vital signs and 12-lead ECG during the study.

## Discussion

This study compared the exposure to levocetirizine when given as levocetirizine OS 5 mg and as cetirizine DS 10 mg under the fasting conditions in healthy Japanese male subjects. The plasma levocetirizine concentration profiles were similar following administration of both preparations. Levocetirizine was absorbed rapidly and subsequently declined steadily with comparable *t*_1/2_ values following administration of levocetirizine 5 mg OS and cetirizine DS 10 mg. The 90% CIs for the geometric mean ratios of the plasma levocetirizine AUC_0–48_ and *C*_max_ were 1.024–1.094 and 0.968–1.091, which were within the range ‘0.80–1.25’ as required to declare bioequivalence in Japanese Guideline for Bioequivalence Studies of Generic Products^13^. Thus, levocetirizine exposure in plasma of the healthy Japanese male subjects following a single oral administration of levocetirizine OS 5 mg was equivalent to that following cetirizine DS 10 mg, suggesting that the pharmacological effects and clinical efficacy of levocetirizine administered as levocetirizine OS 5 mg are similar to those when administered as cetirizine DS 10 mg. A study in non-Japanese male and female healthy subjects^14^ identified statistically significant differences in dose adjusted pharmacokinetic parameters for levocetirizine between male and female subjects, but concluded the discussion by saying that the differences are of no clinical relevance. Although the pharmacokinetics of levocetirizine in females was not investigated in this study, it is unlikely that any clinically relevant differences in pharmacokinetic parameters would be observed between Japanese males and females.

In a previous report^8^, pharmacokinetics after oral administration of levocetirizine 5 mg and 10 mg were investigated in healthy Japanese male subjects. Mean *C*_max_ value was 232.6 ng/mL and achieved within 1.0 h (median) after dosing of a levocetirizine 5 mg tablet. Mean *t*_1/2_ of levocetirizine from the 5 mg tablet was 7.3 h and mean AUC_0–48_ value was 1791.5 ng.h/mL. The pharmacokinetic results in this study are consistent with the findings in the previous study with Japanese subjects. In view of the levocetirizine investigations^5,6,7,15,16^ previously published for clinical safety, efficacy, pharmacokinetics, and satisfaction scores by patients and physicians, levocetirizine OS is a promising, and one of the suitable treatment options for pediatric patients who currently must divide a tablet to relieve the histamine-mediated symptoms associated with allergic disorders.

No AE or clinically significant finding in clinical laboratory tests was reported during the study. Levocetirizine OS 5 mg and cetirizine DS 10 mg were well tolerated in Japanese subjects.

## Conclusion

Levocetirizine exposure in plasma was equivalent when given as levocetirizine OS 5 mg and as cetirizine DS 10 mg. Both preparations were safe and well tolerated in healthy Japanese male subjects.

## Transparency

### Declaration of financial/other relationships

Hiroko Ino, Katsutoshi Hara, Gosuke Honma, and Yohei Doi are employees of GlaxoSmithKline K.K. Hiroyuki Fukase is employed by CPC Clinical Trial Hospital, Medipolis Medical Research Institute, a company that received funding from GlaxoSmithKline to conduct this study. He has received no direct payment for writing or publishing the manuscript.
